# Large Interstitial Pregnancy Treated With Methotrexate: A Case Report and Literature Review

**DOI:** 10.7759/cureus.115914

**Published:** 2026-09-07

**Authors:** Hyeryeon Kang, Toshiyuki Itai, Risa Matsunaga, Etsuko Miyagi

**Affiliations:** 1 Obstetrics and Gynecology, Yokohama City University School of Medicine, Yokohama, JPN

**Keywords:** complication, ectopic pregnancy, interstitial pregnancy, long-term outcome, methotrexate

## Abstract

Interstitial pregnancy (IP) is an ectopic pregnancy in the segment of the fallopian tube within the muscular wall of the uterus. Due to its rarity, the applicability and safety of systemic methotrexate (MTX) administration for IP remain unclear, especially when the mass is relatively large. We encountered a 21-year-old patient who was diagnosed with an asymptomatic IP of 42 mm × 52 mm and beta-human chorionic gonadotropin (β-hCG) of 7,640 mIU/ml. A single dose of systemic MTX at 50 mg/m2 gradually decreased her β-hCG levels, which became undetectable 161 days after treatment; however, the remnant mass persisted. On further follow-up, the mass disappeared 21 months after MTX administration. Our clinical experience suggests that MTX can be considered for large IPs (>4 cm) if the patient is asymptomatic and hemodynamically stable, and that remnant tissue should be recognized as an unignorable complication of MTX treatment for IPs requiring long-term follow-up.

## Introduction

Interstitial pregnancy (IP) is an unusual type of ectopic pregnancy that occurs within the interstitial portion of the fallopian tube and accounts for approximately 2-4% of ectopic pregnancies. Owing to its anatomical location and relatively thick fallopian tube structure compared to other parts, IP has the capacity to expand before rupture more than other tubal pregnancies, leading to later diagnosis, a higher risk of massive hemorrhage, and subsequent mortality [1]. Diagnosis is usually made by ultrasonography; the interstitial line sign, a thin echogenic line connecting the ectopic gestational sac and the endometrial cavity, can be a diagnostic clue [2].

Treatment options for unruptured IPs include surgical and medical treatments. Since the first successful use of methotrexate (MTX) for an IP was described in 1982 [3], systemic or local MTX has been widely used for hemodynamically stable and unruptured ectopic pregnancies as medical treatment [1,4,5]. Relative contraindications include embryonic cardiac activity, high human chorionic gonadotropin (hCG) levels, and the size of an ectopic gestational sac. One systematic review reported a higher failure rate of MTX treatment for ectopic pregnancies when the initial hCG was > 5,000 mIU/mL [6]. Furthermore, a gestational sac greater than 4 cm is a relative contraindication for MTX treatment [4], and most reported IPs treated with MTX were less than 4 cm [7]. However, given the expansibility of the interstitial portion, it might be reasonable to consider MTX treatment for large IPs, greater than 4 cm.

Here, we report a 42 × 52 mm IP with an initial β-hCG of 7,640 mIU/ml that was successfully treated with systemic MTX. Although her β-hCG levels decreased smoothly after treatment, it took 21 months for the ectopic mass to disappear. Together with other reported IPs greater than 4 cm, which is slightly greater than 2 standard deviations (SD) among reported IPs treated with MTX [7], it is suggested that MTX for large IPs can be practicable if the patient understands its risks and caveats, which require close monitoring during the first few weeks and possibly long-term follow-up even after β-hCG becomes undetectable.

## Case presentation

The patient’s clinical course, including the trend in β-hCG levels and images of ectopic masses, is summarized in Figures 1-3. The patient was a 21-year-old woman, gravida 2 para 0. She had a history of abortion, but her medical history was otherwise unremarkable. 

**Figure 1:**
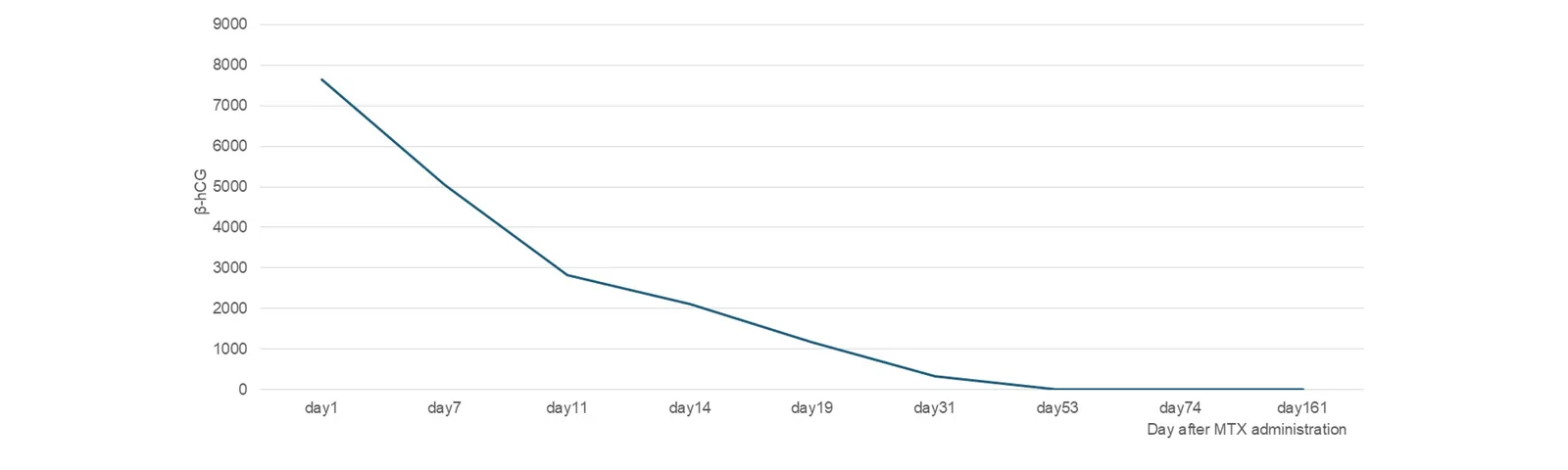
The beta-human chorionic gonadotropin (β-hCG) levels of the patient. MTX: methotrexate

**Figure 2:**
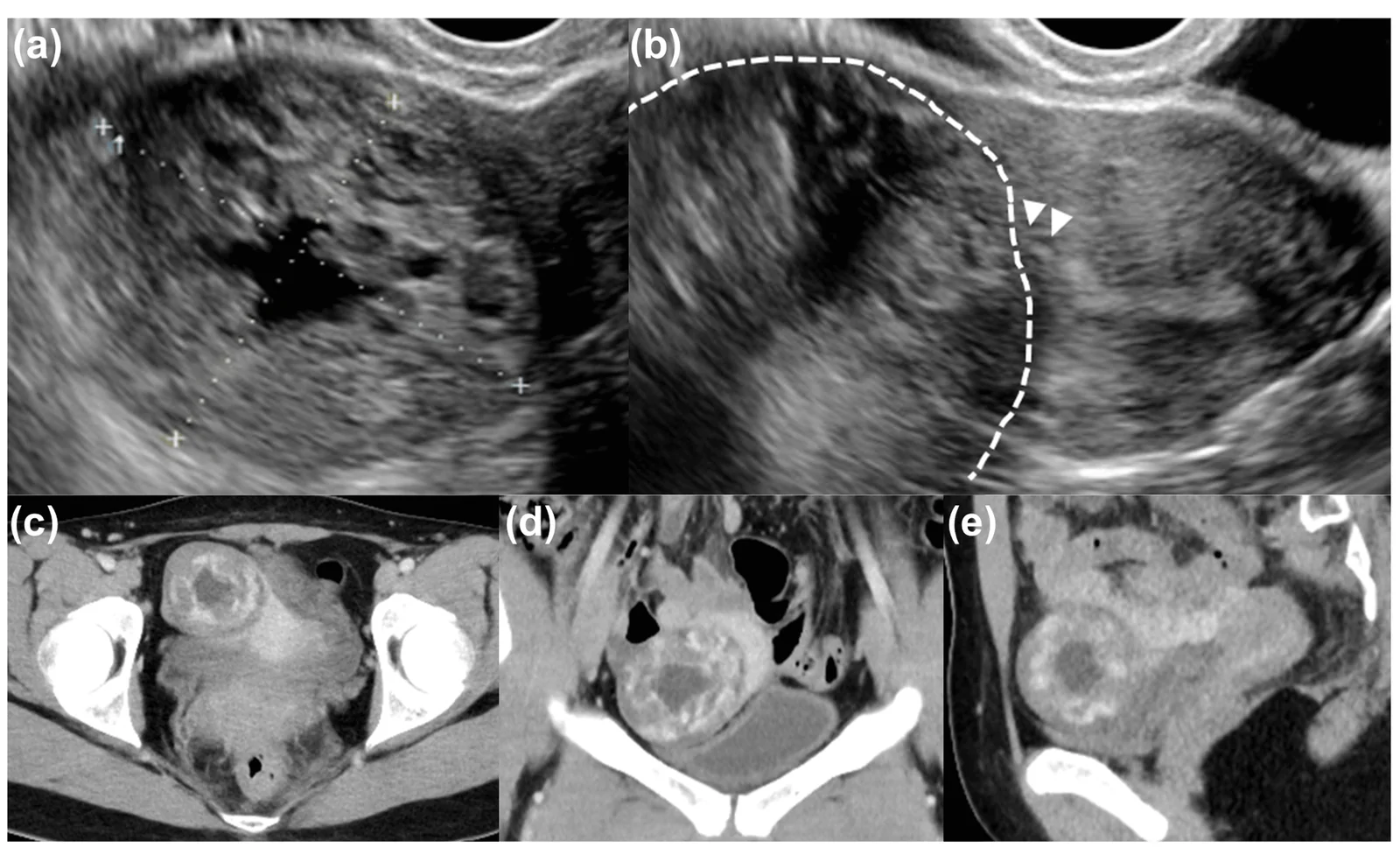
Imaging studies before treatment. (a) Ultrasonography of a 5-cm ectopic gestational mass. (b) The mass (dotted line) lies outside the endometrium, with an interstitial line (arrowheads). Contrast-enhanced CT scans before treatment are consistent with ultrasonography findings. (c) Axial. (d) Coronal. (e) Sagittal.

**Figure 3:**
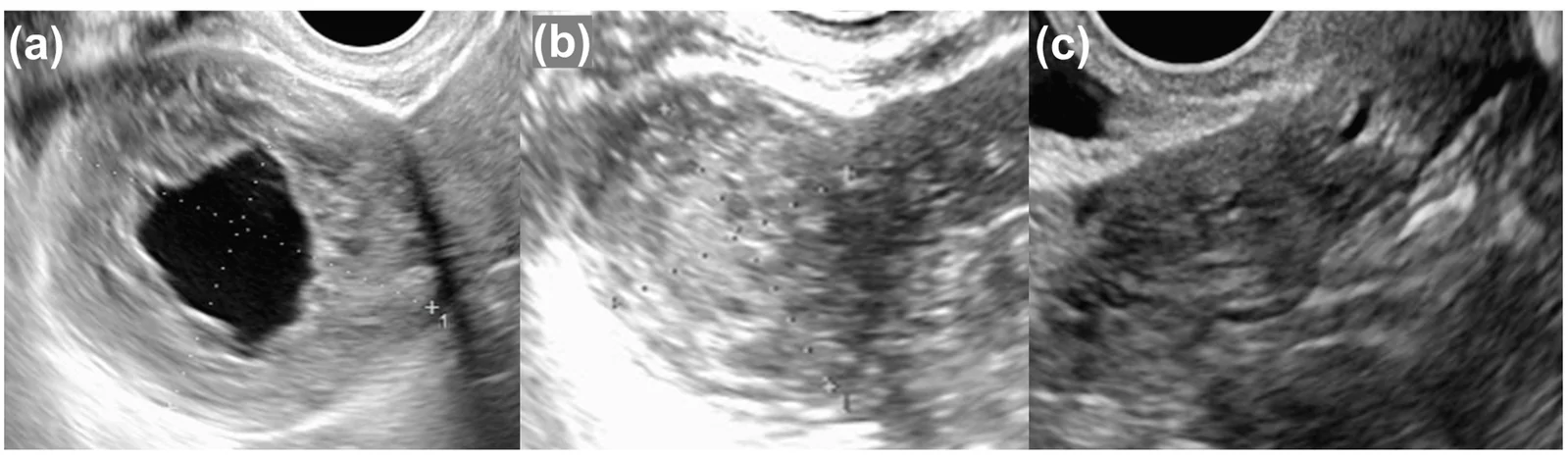
Ultrasonography of the ectopic gestational mass after the MTX treatment. (a) Two months after treatment (61.0 × 52.8 mm). (b) One year after treatment (33.9 × 28.7 mm). (c) Twenty-one months after treatment. The mass is undetectable. MTX: methotrexate

She visited our institution for a six-week amenorrhea, and the pregnancy test was positive. Her β-hCG level was 7,640 mIU/mL (Figure 1), and ultrasonography revealed a 5-cm mass in the interstitial part with an interstitial line (Figure 2A, 2B), with which an IP was suspected. As she did not opt to continue her pregnancy, contrast-enhanced computed tomography was performed to further detail the anatomical location of the ectopic pregnancy, which revealed a 51-mm mass lesion in the right fallopian tube angle with a thick ring-shaped contrast effect (Figure 2C-2E). No endometrial thickening was noted. Based on these findings, the patient was diagnosed with an IP. MTX was chosen for her treatment for the following reasons. First, her β-hCG level had not increased significantly over the past few days, and a fetal heartbeat had not been detected. Second, she was hemodynamically stable without any episodes of vaginal bleeding. Third, given its location, we considered laparoscopic surgery unsuitable due to potential complications.

She received a single dose of systemic MTX (50 mg/m2). On days four and seven, her β-hCG levels were 6,424 and 5,043 mIU/mL, respectively; the drop rate was over 15%; therefore, she did not receive an additional MTX dose, and her β-hCG levels were carefully monitored. Her β-hCG levels decreased smoothly and became undetectable 161 days after treatment (Figure 1). The ectopic gestational mass enlarged up to 61.0 × 52.8 mm two months after MTX treatment (Figure 3A) and then gradually decreased in size. However, the mass remained (Figure 3B) one year after the MTX treatment (6.5 months after her β-hCG level became undetectable). The patient was advised to avoid becoming pregnant. On further follow-up, the mass disappeared 21 months after the initial MTX treatment (Figure 3C). The patient received clearance to conceive but was advised to visit our department once her pregnancy test was positive in case of a recurrent ectopic pregnancy, where the mass resolution occurred.

## Discussion

We report a woman diagnosed with an IP of 5 cm, which resolved after a systemic MTX dose but required a long-term follow-up due to the remnant tissue after the treatment. As there are no established criteria for MTX in IPs, particularly regarding their size and applicability, our management was based mainly on clinical evaluation and the available reports. Our clinical experience suggests that systemic MTX may be feasible even with a relatively large mass if the patient is asymptomatic and hemodynamically stable, and that a remnant mass requiring long-term follow-up is an unignorable complication of the treatment.

A recently published systematic review of 124 patients with IP reported a success rate of approximately 70% for MTX treatment [7]. In their study, the mean ± SD of the mass size was 18.1 ± 10.4 mm, and patients with baseline β-hCG >5,000 mIU/mL had higher rates of failure and complications. However, it did not explain how to interpret IP sizes in relation to treatment choice. We manually searched previous publications; 18 large IPs that were successfully treated with MTX have been reported [8-17], including a 6-cm IP patient (No. 9) with a positive fetal heartbeat and a β-hCG level of 106,634 mIU/mL (Table 1). Two patients (No. 4 and 5) had pelvic pain, and all patients were hemodynamically stable. In 15 patients, the β-hCG levels exceeded 5,000 mIU/mL. Taken together, MTX treatment may be considered regardless of mass size, especially when β-hCG levels are not significantly elevated, provided the patient understands the benefits and risks of the treatment and is closely monitored to assess its efficacy.

**Table 1:** Reported patients with interstitial pregnancy whose ectopic masses were greater than 4 cm. *No. 2 was diagnosed with heterotopic interstitial pregnancy. β-hCG: beta-human chorionic gonadotropin, CRL: crown-rump length

No	Reference	Patient ID	Age	Parity	Mass size	β-hCG (IU/L)	Resolution time	
							Mass	b-hCG
1	Our patient	-	early 20s		42 x 52 mm	7640	21 months	161 days
2	Li et al. [10]	-	31	NA	58 x 40 mm	264000*	200 days	NA
3	Galani et al. [8]		35	G2P1	40.8 mm (CRL)	60530	6 weeks	9.3 (6 weeks)
4	Uludag et al. [9]	1	29	G1P0	44 mm	30605	10 weeks	7 weeks
5	Uludag et al. [9]	3	27	G3P2	46 mm	72645	14 weeks	12 weeks
6	Uludag et al. [9]	10	28	G1P0	44 mm	53450	12 weeks	8 weeks
7	Corioni et al. [16]	-	36	G2P1	46 x 37 mm	8681	1 year	2 months
8	Tanaka et al. [11]	1	-	-	5 cm	2466	-	30 days
9	Tanaka et al. [11]	4	-	-	6 cm	106634	-	129 days
10	Tanaka et al. [11]	8	-	-	4 cm	36000	-	55 days
11	Tanaka et al. [11]	29	-	-	4 cm	610	-	26 days
12	Wright et al. [12]	1	27	G2P0	72 x 48 x 61 mm	17180	-	12 weeks
13	Jermy et al. [13]	3	20	P0	45 x 46 x 47 mm	7168	-	55 days
14	Jermy et al. [13]	7	29	P1	42 x 32 x 34 mm	31381	-	94 days
15	Jermy et al. [13]	19	27	P0	43 x 52 x 54 mm	580	-	80 days
16	Sagiv et al. [17]	1	35	P2	55 x 40 mm	15400	4 months	4 months
17	Hajenius et al. [14]	3	20	P1	43 mm	81000	635 days	148 days
18	Hajenius et al. [14]	6	28	P1	40 mm	15600	119 days	120 days
19	Hoek et al. [15]	-	34	G2P1	40 mm	70600	-	197 days

The remnant mass after MTX treatment necessitated long-term follow-up in our patient. Of the 18 patients reported (Table 1), two patients (No. 7 and 17) required long-term follow-up to confirm mass resolution for one year or longer. Including our patient, three out of 19 patients (15.8%) experienced this complication. There were a few case reports of remnant tissues in tubal pregnancies after MTX treatment [18-20], which were pathologically diagnosed with hematocele or mummified fetal tissues and surgically repaired. There have been no IP patients who underwent surgery for remnant mass after MTX treatment. Given that one patient (No. 16) had a recurrent IP that required surgery after the successful resolution of the ectopic mass four months after MTX treatment, we consider that patients with a remaining mass after MTX treatment are at risk for recurrent ectopic pregnancies.

## Conclusions

In conclusion, our patient’s clinical course and the literature review suggest that MTX could be considered for IP with masses greater than 5 cm, but it requires close monitoring and long-term follow-up, as a remnant mass may occur in approximately 16% of these cases. Future studies should collect more IP cases with MTX treatment, especially failure cases, to establish criteria for MTX treatment and its management protocol based on detailed information on short- and long-term complications of MTX.
